# Author’s Reply to: Environmental Influence and Recruitment Bias in Studies on Internet Addiction. Comment on “Addiction Symptom Network of Young Internet Users: Network Analysis”

**DOI:** 10.2196/45607

**Published:** 2023-07-11

**Authors:** Hui Zheng

**Affiliations:** 1 Shanghai Key Laboratory of Psychotic Disorders Brain Health Institute, National Center for Mental Disorders, Shanghai Mental Health Center Shanghai Jiao Tong University School of Medicine Shanghai China

**Keywords:** internet addiction, Internet Addiction Test, network analysis, adolescents

## Letter to the Editor

Huang and Liaw [1] have raised some valid concerns regarding the interpretation of the results of our paper on internet addiction [2]. Specifically, they have suggested that the lack of detail in the paper's methodology may reduce confidence in the findings. In addition, there is a possibility that environmental factors such as network availability and potential bias in advertising recruitment methods had a substantial influence on the results. To bolster this research and increase its robustness, it is important to provide greater clarity and engage in further discussion to address these issues [3].

We concur with the importance of environmental factors in the phenomena of internet usage behavior becoming pathologic and maladaptive. Nevertheless, this process could be elucidated through the interplay between biological (eg, genetic and neurobiology) and environmental (eg, social and natural) factors under the bio-psycho-social model. However, our study did not focus on the development and maintenance process. Specifically, our study did not consider the ease of access to internet cafés and the outdoors, the degree of urbanization, etc. We provide a 2-fold response to address this.

First, it is unlikely that the presence of internet cafés contributes to maintaining IA as participants were college students who mostly accessed the internet via smartphones rather than computers. Therefore, the pattern of mobile phone usage should be an important topic in digital health, such as whether specific mobile phone apps are directly related to internet addiction. Second, despite ongoing pandemic-related restrictions, outdoor activities at the 2 time points when data were collected mean that subjects were allowed to do outdoor activities on a daily basis, but not outside of the school in a semi-restricted situation. Since sample acquisition was derived from a single center, it is not feasible to study urbanization or ease of access to outdoor activities. We believe such factors should be addressed through longitudinal developmental research such as the Global Imaging Genetics Initiative in Adolescence [4], coupled with geographic information [5]. Combining biological samples, neuroimaging data, self-reported questionnaires, and socioeconomic records could be one feasible approach to investigate how they affect mental health outcomes.

With regard to how recruitment via advertising was implemented, a plethora of prior studies has contended that online recruitment is an effective strategy [6], which justifies its use in our paper. We employed a three-step procedure: (1) the content of the research was posted on the university’s bulletin board system, making it accessible to all students who can then voluntarily participate; (2) after 1 to 2 weeks, we distributed information to current students via SMS text messaging or social media apps (WeChat or QQ) through a counselor who invited students to voluntarily participate; and (3) out of those who consented electronically or signed informed consent forms (provided pictures of their handwritten consent to participate), a strict data quality control process (ensuring the response time was above the minimum threshold, the response mode passed verification, the polygraph questions were answered correctly, and self-reported involvement was high) ensured that 50% of responses were incorporated in the final analysis. We plan to continue using an improved iteration of this process going forward. This technique may potentially introduce bias, but it is efficacious for mediating between student motivation for participating and collecting meaningful data efficiently.
